# Electricity and Galvanism Explained on the Mechanical Theory of Matter and Motion

**Published:** 1820-11

**Authors:** Richard Phillips


FOR THE LONDON MEDICAL AND PHYSICAL JOURNAL.

### *Electricity and Galvanism explained on the Mechanical Theory of Matter and Motion.* By Sir Richard Phillips.

In no branch of philosophy have superstition and the love of the marvellous revelled in greater luxury of variety and absurdity, than in every existing disquisition, observation, and theory, of the classes of phenomena called Electrical.

Theories assuming miraculous principles which never had existence, and which are inconsistent with that Supreme power for whose support they were weakly invented, and then a course of reasoning by false analogies, have led to all these absurdities. The *philosophical* electrician talks flippantly of his fluids and his fires,—his negatives and his positives,—his charges, surcharges, and discharges,—his saturations and non-saturations,—his attractions and repulsions,—and other conjurations; and believes that he can bottle-up this fluid *sui generis;* that a cloud can be surcharged with it; that bodies contain more or less than their natural quantity; and a hundred other equal errors. It is therefore to be feared, that he will be as much enraged at the writer of this, as the devotees of the gravitating effluvia and of the eternal projectile force, on its being proved that there is, in truth, no such thing as an electrical fluid, and that all the appearances are mere mechanical accidents of passive matter temporarily disturbed by the causes which generate electrical phenomena.

We shall not be long in arriving at this conclusion; but we must look to *facts,* and not to *theories;* and must avoid false analogies founded on erroneous theories.

*Fact* 1.—Every exhibition of electrical phenomena takes place in and within electrics only.

*Fact* 2.—The condensation or accumulation of force takes place at the surface of any body which bounds the electric.

*Fact* 3.—No force appears at one surface of an electric, unless a similar force appears at the opposed surface.

*Fact* 4.—The force at one surface is of a contrary character to that at the opposed surface.

*Fact* 5.—The force at one surface has an oxygenating or acid effect, and that at the opposed surface an azotic or alkaline effect.

*Fact* 6.—In the galvanic excitement a palpable decomposition takes place in the fluid lying between the plates, and the latent elements decomposed appear in the acid and alkali at the opposite ends of the series.

*Fact* 7.—In the excitement of glass and all other electric plates, one side of the plate becomes simultaneously in an opposite state to the other, and must therefore be of suitable thickness, and in circumstances permitting its corresponding change of state, and the opposed sides exhibit respectively the acid and alkaline properties.

*Fact* 8.—It makes no difference whether the conducting surface which bounds the electric, be thick or thin; whether it be a solid metal or gold leaf.

*Fact* 9.—When the electric plate, as such, is destroyed by the interposition or continuity of any non-electric, or conductor, an equilibrium takes place within the disturbed electric; and the opposed surfaces of the electric cease to exhibit electrical phenomena.

*Fact* 10.—When the opposed surfaces are brought near to each other, an equilibrium takes place by a spark which proceeds from any projecting point of one of the surfaces; and heat and light are elicited.

*Fact* 11.—Unless the parts of each opposed surface are united, or rendered continuous by a conductor, the phenomena are inconsiderable.

*Fact* 12.—Some mechanical action, as friction, variation of volume, or different power of receiving or radiating heat, or atomic motion, is necessary to the production of electrical phenomena.

*Fact* 13.—The power, when excited in any electric, is capable of being transferred to any other electric, provided the surfaces be coated with a conductor.

*Fact* 14.—Bodies are conductors nearly in the same ratio as that in which they are conductors of heat or atomic motion; and they are electrics by a contrary law.

*Fact* 15.—All electrical phenomena take place, and all successful experiments are made, within atmospheric air.

*Deductions from these Facts.*—Fact 8, proves that electricity does not permeate the substance of conductors; for, whether a conductor is hollow or solid, or of glass, or baked wood covered with gold leaf, or of solid metal, the effect is equally powerful. It is therefore a gross error to speak of conducting bodies as charged or surcharged, or as containing more or less than their natural quantity, &c.

Fact 1, proves that the electrical power resides within the adjoining electric; and this fact, and Fact 8, prove that it does not reside within the body of the conductor; the best conductors having no conducting substance, but only a conducting surface.

Fact 15, proves that air is the universal electric, and that in all cases a plate of air is the thing affected. But air is composed of twenty or twenty-one volumes of oxygen, and eighty or seventy-nine volumes of azote; being the very principles which, by Facts 5, 6, and 7, are evolved and extricated on the opposed sides of every electric plate.

It is inferred from Facts 5, 6, and 7, that all cases of electrical excitement consist merely of the decomposition or separation of the acid and alkaline principles natural to the substance and constitution of the body, or electric plate; and that the various phenomena attending the partial or general restoration constitute all the appearances called electric and galvanic.

Is the electric power, therefore, any thing more than a mechanical separation, or decomposition, of the constituent or gazeous portions of the electric? Are not the gazeous portions, by some peculiar motions, carried to the positive and negative sides of the plate? Do not all the phenomena proceed, first, from the endeavour of the oxygen and azote to return to their fit combination in air; and, second, from this often taking place suddenly? Is electricity, in fine, any thing more than an accident of the constituent atoms of air, or the similar atoms of other electrics? Or, in other words, as all experiments and phenomena take place in air, are not the phenomena of all other electrics merely relative to the powers of air, and governed by the relative powers of each to the other, as partial conductors and partial electrics?

These questions, and the consideration of all the facts, lead to the general conclusion, that there is no fluid *sui generis* producing electrical phenomena, nor any peculiar fluid, nor any fluid whatever, concerned in electrical phenomena; and that all this class of phenomena arises from the mechanical decomposition, or temporary separation, of the constituent elements of the atmospheric air, or electric medium or fluid, interposed between conducting surfaces; within which electrics all the phenomena take place, as well between them and conductors, as between them and other adjacent electrics, and between them and other electrics and conductors.

Electricity is therefore an accident of air, or of the atoms of air, just as wind is an accident of air in mass; and it would be as rational to refer a storm to a peculiar fluid, as it is to refer the phenomena called electrical to a peculiar fluid.

But at the time when the peculiar fluid was first invented, the constituent parts of air had not been discovered; just as the two-fold motions of the earth were not suspected, when the fall of bodies was superstitiously ascribed to the earth's attraction; or just as the rotation of the earth round the fulcrum of the earth and moon was not suspected, when the tides were superstiously ascribed to the attraction of the moon. But new facts and improved reasoning render it highly proper to get rid of all these properties *per se*, fluids *sui generis*, and attractions without mechanical cause.

The excitement, whatever it be, is mechanical, and it produces the mechanical effect of separating the constituent atoms of an affected or electrified plate of air, or other electric.

If we excite glass, &c. we produce a preponderance of the acid or oxygen atoms of the proximate surface of air, and the withdrawing of these necessarily occasions an apparent preponderance of alkaline or azotic atoms on the opposed surface which it has hitherto been so difficult to understand.

If we coat the glass surface with a conductor, or congeries of atoms more capable than glass or air of conducting heat or atomic motion, we then unite or connect the points of the electric plate or plate of air.

If we present a similar coated surface in opposition to the first excited plate, we then produce a maximum of effect, *i. e.* two surfaces which unite all the points of the surfaces of the plate of air; one of which is oxygenated, or positive, and the other azotic, or negative; both exerting considerable force to co-mix in that state of fitness which rendered them atmospheric air.*

If then any light body, or body whose inertia is less than the force with which the atoms seek to re-unite, be presented between the surfaces, or to one surface, (the other being supposed or understood, and existing in the hand, the operator, or the walls,) then the said body will be driven or apparently attracted, and will assist in restoring the equilibrium of the affected electric plate.

If the surfaces be moved so near that the excitement which separated the atoms is overcome by the aptitude of their forms to re-unite, and if any small point project on either surface, carrying the surfaces nearer by the thickness of the said point, then the re-union of the entire surface takes place through that point, and the concentrated force of the simultaneous rush of the oxygenous atoms in one direction, and the nitrogenous atoms

* Thirty-two years ago the writer made his prime conductor of a board covered with tin-foil, and, adopting the principle that every conductor is, in fact, but a coating to a plate of air, he arranged similar boards above and below, and thereby decomposed a double plate of air. Galvanism was then unknown; but, if he had heard of the voltaic pile, he would certainly have imitated it in a common electrical circle. He conceives that the *accelerated power* gained in this way, would be far more splendid than in the galvanic circle; because, in electricity, the power is expanded, and results from the energy of natural restoration; but, in galvanism, the exciting power is limited, and not restored, but dissipated.

in the opposite, produces the action called *light;* and also mechanical effects on all bodies which contain either oxygen or nitrogen.

The restoration, the double current, the spark, (the stream being an optical illusion,) and most of the other wonders, vanish therefore, when examined by a rational mechanical theory.

The great phenomena of nature which take place when a vast affected plate of the atmosphere is coated with clouds, are easily understood. Some exciting cause, generally the atomic motion of heat, has decomposed the air; but the effects are dissipated in space, till a cloud coats the upper surface, and connects all the points of the affected plate of the atmosphere. Under these circumstances, some cloud or point of a cloud, sinking below the general level of the surface, or some projecting point on the earth, narrows the plate in that place, and a concentrated restoration, or partial restoration, takes place at that point, exhibiting lightning, &c.

One might trace, examine, and easily explain, all the details of the phenomena on this simple and natural theory, but enough has been said to show that *electricity* is no exception to the mechanical principles of matter and motion; and, in regard to the kindred phenomena of *galvanism*, I will content myself with observing, that it is merely *accelerated electricity;* the interposing fluid being palpably decomposed and evolving the electrical powers, each term in the series of plates being a new impulse or power added to the previous one, till the ultimate effect is accelerated, like that of a body falling by the continuous impulses of the earth's motions, or like a nail heated red-hot by accelerations of atomic motion produced by repeated percussions of a hammer.

*London; July* 11, 1820.

